# Medicinal Employment of Gutta Percha

**Published:** 1848-09

**Authors:** 


*Medicinal Employment of Gutta Percha.*—Gutta Percha being very soluble in sulphuret of carbon, and the latter not losing any of its great volatility by the combination, M. Uytterhoeven, head surgeon of St. John's Hospital, in Brussels, is in the habit of spreading the mixture in its liquid state over parts which he is anxious to preserve from the action of air and water. Recently, an abscess resulting from caries of the ribs, after being emptied, was covered by a coat of this fluid, a small patch of court-plaster having previously been placed on the puncture. In this manner the inflammation of the parietes of the cyst was prevented, and the latter was emptied as often as accumulation of matter required. The same surgeon has employed the material, prepared in the manner mentioned above, to prevent the entrance of air into an articulation laid open by a wound. There is no doubt that chloroform might advantageously replace the sulphuret of carbon.—*Lancet.*

